# Graphene with Ni-Grid as Semitransparent Electrode for Bulk Heterojunction Solar Cells (BHJ-SCs)

**DOI:** 10.3390/polym14051046

**Published:** 2022-03-05

**Authors:** Martina Dianetti, Gianpaolo Susanna, Emanuele Calabrò, Giuseppina Polino, Martin Otto, Daniel Neumaier, Andrea Reale, Francesca Brunetti

**Affiliations:** 1 Department of Electronic Engineering, University of Rome Tor Vergata—CHOSE, Via del Politecnico 1, 00133 Rome, Italy; martina.dianetti@uniroma2.it (M.D.); gianpaolo.susanna.ext@mise.gov.it (G.S.); emanuele.calabro@uniroma2.it (E.C.); giuseppina.polino@uniroma2.it (G.P.); reale@ing.uniroma2.it (A.R.); 2 ISCTI—Istituto Superiore delle Comunicazioni e delle Tecnologie dell’Informazione-Ministero dello Sviluppo Economico, Viale America 201, 00144 Rome, Italy; 3 Gesellschaft für Angewandte Mikro-und Optoelektronic mBH—AMO GmBH, Otto-Blumenthal-Straße 25, 52074 Aachen, Germany; otto@amo.de (M.O.); neumaier@amo.de (D.N.); 4 Chair of Smart Sensor Systems, Bergische University of Wuppertal, 42119 Wuppertal, Germany

**Keywords:** graphene based solar cells, TCO-free solar cells, chemical vapour deposition (CVD), low band-gap, bulk-heterojunction solar cells, Ni-grid/MLG

## Abstract

In this work, we present the fabrication and characterization of bulk-heterojunction solar cells on monolayer graphene (MLG) with nickel-grids (Ni-grid) as semitransparent conductive electrode. The electrodes showed a maximum transmittance of 90% (calculated in 300–800 nm range) and a sheet resistance down to 35 Ω/□. On these new anodes, we fabricated TCO free BHJ-SCs using PTB7 blended with PC70BM fullerene derivative as active layer. The best device exhibited a power conversion efficiency (PCE) of 4.2% in direct configuration and 3.6% in inverted configuration. The reference solar cell, realized on the ITO glass substrate, achieved a PCE of 6.1% and 6.7% in direct and inverted configuration respectively; for comparison we also tested OSCs only with simple Ni-grid as semitransparent and conductive electrode, obtaining a low PCE of 0.7%. The proposed approach to realize graphene-based electrodes could be a possible route to reduce the overall impact of the sheet resistance of this type of electrodes allowing their use in several optoelectronic devices.

## 1. Introduction

In the last two decades, organic solar cells (OSCs) have seen a growing interest in photovoltaic research due to their versatility as printable low-cost electronics with low weight, flexibility, low temperature processing, large area coating and roll-to-roll applicability coupled with high power conversion efficiency (PCE) [1]. The research effort of the last two decades in materials, techniques, processing, device architecture, morphology control and interface engineering with new non-fullerene polymer materials, has achieved a PCEs above 18%, reducing the gap to the well-established silicon technology [2,3,4,5]. The most common material used as transparent conductive oxide (TCO) in OSCs is indium tin oxide (ITO); however, beside it’s high transparency and conductivity (more than 80% and 15 Ω/□ respectively), there are several critical issues: (i) limited indium sources, (ii) high cost due to the deposition techniques (sputtering, pulsed laser deposition and electroplating, etc.), (iii) high temperature processing, (iv) high mechanical brittleness and (v) low transparency in the violet region [6,7]. For these reasons, several materials, such as conductive polymers, carbon nanotubes, silver nanowires, metal grids and graphene, have been investigated as alternative TCO for photovoltaic applications [8,9,10,11,12]. The most promising carbon-based materials is the graphene, a two-dimensional layer of carbon atoms covalently bonded in a hexagonal lattice structure. Graphene has gained significant attention in the field of optoelectronics due to its outstanding electronic, optical, thermal and mechanical properties [13,14]. Different synthesis techniques have been used to obtain graphene films independently from the substrate types, including mechanical exfoliation of graphite crystals, chemical exfoliation and reduction of graphene oxide, and growth by chemical vapor deposition (CVD) [15,16,17]. The CVD method of graphene growth offers several advantages for TCO fabrication due to the facility of growing high quality, continuous and uniform films on large areas with low sheet resistances, a highly controlled tunability of the synthesized film properties and the transferability to flexible or rigid substrates [18,19]. Typically, graphene grown by CVD has a sheet resistance (RSHEET) between 500 Ω/□ to 1000 Ω/□ and a mobility of 300–4000 cm^2^/Vs [20]; these values are suitable for many electronic applications, such as smart windows, field emission transistors, sensors or storage applications; however, for the above-mentioned application as TCO the RSHEET still remains an order of magnitude too large [21]. Nowadays, several methods for reducing the graphene RSHEET have been developed using chemical dopants, such as UV/O_3_ or chlorine surface doping or silver (Ag) grid [22,23,24,25]. One of the first graphene-based OSCs was developed in 2008 by Wu et al., by using a single-graphene layer (SLG) as electrode deposited by solution-processing on quartz substrates from aqueous dispersion [26]. The devices fabricated on such transparent conductive electrodes (TCE) achieved an efficiency of only 0.4%, the half respect the ITO-based counterpart. The strategy to dope the graphene-electrode to decrease the RSHEET by increasing the number of charge carrier through acid additives was shown by Wang et al. in 2011. By surface modification of a thin MoO_3_ layer on top of a 4-layer graphene stack, the authors achieved a PCE of 2.5%, close to the 3% obtained on the ITO substrate [27]. Further PCE enhancement of the graphene-based ITO-free OSCs has been obtained in 2014 by employing silver grids on top of the graphene layer, achieving a PCE of 2.9% on flexible substrates [24]. In 2018, La Notte et al. fabricated fully-sprayed flexible semitransparent OSCs by employing CVD graphene top electrode modified with cellulose additive via lamination process, achieving PCEs over 3% in a single cell and almost 1% in mini-modules [28,29]. Today, graphene based OSCs have reached very significant high PCE that makes this a promising material [30,31,32,33,34].

In this work, we report the application of monolayer graphene (MLG) coupled with nickel (Ni)-grids on quartz substrates. Ni-derivatives were recently employed with graphene-layers to form hierarchical nanocomposites as sustainable counter electrodes for pt-free dye sensitized solar cells (DSSC) [35] and are under study for electromechanical and flexible electronics [36,37,38]. Ni has a very low contact resistance to graphene, so it is a good candidate to collect charges from the semitransparent graphene electrode [8]. Here, the use of a Ni-grid has been evaluated for the first time in combination with MLG in order to realize organic photovoltaic. Graphene films synthesized by thermal CVD were transferred on top of Ni-grids that were realized with a specific geometric profile to avoid cracks in the thin graphene layers and were used as transparent anode to fabricate OSCs. The direct and the inverted architectures have been realized on MLG transferred on Ni grids and, thanks to the very low contact sheet resistance of Ni to graphene, the devices reached a maximum PCE of 4.2% and 3.6%, respectively, showing the possibility to use Ni/graphene as an efficient electrode for OSCs.

## 2. Materials and Methods

### 2.1. Preparation and Characterization of GRAPHENE on Ni-Grid as Electrode

**Figure 1:**
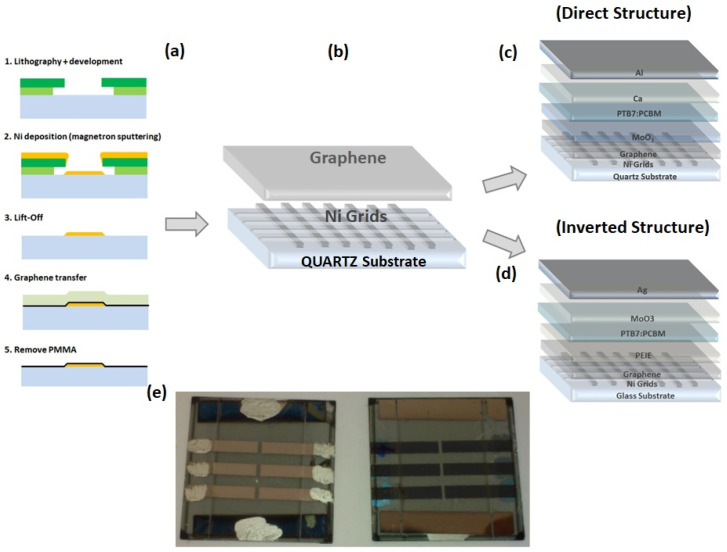
(**a**,**b**) Fabrication process of graphene/grid substrates: the lithographic step defines an undercut on the photoresist, the deposition of the nickel, followed by the lift off process defines the grid, then the graphene layer previously transferred on PMMA layer is deposited on the grid creating the electrode final electrode. (**c**) Direct solar cells on graphene grids; (**d**) Inverted solar cells on graphene grids; (**e**) Final layout of a BHJ-SCs PTB7-based on graphene/grid substrate (front and back view).

**Figure 2:**
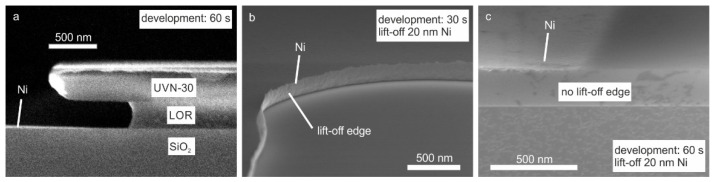
(**a**) Cross section SEM image of a sample after lithography and metal deposition before lift-off. (**b**) Example of a lift-off edge due to an insufficiently large undercut. (**c**) Example of a grid line without a lift-off edge.

**Figure 3:**
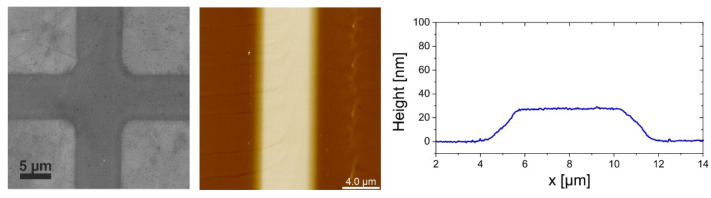
SEM (**left**) image of the metal grid covered with graphene and AFM (**center**) image along with an AFM profile (**right**) that shows the shallow slope of the lines.

The Ni-grids were deposited on quartz substrates via sputter deposition and conventional optical lithography followed by lift-off. For lithography, a 2-layer resist was used to create an undercut large enough to avoid metal lift-off edges during the metal deposition. The bottom layer was LOR-3A, which is insensitive to light but soluble in developer, even without exposure. The top layer was the negative resist UVN-30. After exposure and post-exposure bake, the UVN-30 was developed in the developer MF26A. The developer also dissolved the LOR3A, while not attacking the exposed regions of UVN30. Thus, the length of the undercut could be controlled by the development time. After lithography, nickel was deposited by magnetron sputtering followed by a lift-off to remove the resist. For this technological process, whose steps are reported in Figure 1a, it was necessary to achieve an undercut of at least 600 nm to avoid lift-off edges, which could damage graphene deposited on top of the grid (Figure 1b). The importance of the undercut can be seen in Figure 2. In Figure 2a, the cross-section SEM image of a sample after lithography and metal deposition is shown for a development time of 60 s. The undercut in this case was around 800 nm. To avoid lift-off edges, an undercut of 600 nm is required. The SEM image in Figure 2b shows the metal grid after lift-off for a development time of 30 s. The undercut was not sufficiently large, so that nickel was deposited onto the sidewall of the LOR-3A layer. These lift-off edges were thinner than the deposition thickness and could be up to 200 nm high, possibly causing short circuits in the solar cell that was fabricated on top of the electrode. With a sufficiently large undercut, lift-off edges are completely avoided, as seen in Figure 2c. A top-view SEM image of a section of metal grid after graphene deposition can be seen in Figure 3, along with an AFM image of a grid line and the corresponding profile of the line. The sidewalls of the lines are very flat, rising slowly over a distance of 1 μm to the final thickness of the metal line. This is a result of the sputtering process and has the advantage of avoiding steep and sharp edges which could damage the graphene that is deposited on top of the grid.

Ni-grids with square sizes of 75 µm × 75 µm, 150 µm × 150 µm, 200 µm × 200 µm, and 300 µm × 300 µm and a thickness of either 20, 40, or 80 nm were fabricated with a constant line width of 5 µm. Commercial graphene grown on copper foil by thermal CVD was transferred onto Ni grid/quartz substrates. The transfer was carried out by wet chemical etching of the copper using PMMA as a mechanical support for the graphene similarly to the method described in Ref. [39]. The sheet resistance of the electrode was measured using a four-point-probe system with a current sourcemeter (Keithley 2420, Cleveland, OH, USA) and Jandel cylindrical probe. Optical transmittance was measured using a Shimadzu UV-2550 (PC)/MPC 2200 spectrophotometer with an integrating sphere. The values of the transmittance in this paper include the absorption of the quartz substrates.

### 2.2. Realization of the Solar Cells

References solar cells devices were made up on ITO glass-covered substrates (Kintec 15 Ω/□) patterned with wet-etching in hydrobromic acid and cleaned in an ultrastrasonic bath with acetone and ethanol (10 min each step). Subsequently, samples were transferred inside the glove box with controlled nitrogen (N_2_) atmosphere. Direct architecture was tested with evaporated molybdenum (VI) oxide (MoO_3_, 99.98% powder Sigma Aldrich, St. Louis, MO, USA) as hole transporting layer (HTL) on top of the semitransparent electrode and a bilayer of evaporated calcium (Ca) and aluminum (Al) as top electrode (Figure 1c). The inverted architecture had Polyethylenimine-ethoxylated (PEIE) as electron transporting layer (ETL) in contact with the semitransparent electrode and a bilayer of MoO_3_ silver (Ag) as top electrode (Figure 1d). The PEIE was dissolved in 2-Methoxyethanol (0.4 wt%), spin coated at 4000 rpm for 60 s and then dried in air at 120 °C for 20 min.

We used Poly[[4,8-bis[(2-ethylhexyl)oxy]benzo[1,2-b:4,5-b0]dithiophene-2,6-diyl][3-fluoro-2-[(2ethylhexyl)carbonyl]thieno[3,4-b]thiophenediyl]] (commonly known as PTB7) blended with [6,6]-Phenyl-C71-butyric acid methyl-ester (PC70BM fullerene derivative). PTB7 and PC70BM (99.99%) were purchased from Solarmer and Solenne BV respectively and combined in a ratio of 1:1.5 and dissolved in 2.2 wt% in chlorobenzene (Sigma Aldrich). Finally, the 3 vol% of 1,8-diiodooctane (DIO) was added in the solutions to improve the morphology of the blend [40]. The active blend was spin-coated at 1000 rpm for 120 s, and then kept in slight vacuum (10^−1^ mbar) for 20 min to accelerate the drying process and remove residual DIO from thin films. Finally, the samples were introduced into a high vacuum chamber (1 × 10^−6^ mbar) in order to evaporate Ca/Al back contacts (5 nm and 100 nm of thickness respectively) or MoO_3_/Ag back contacts (5 nm and 100 nm of thickness respectively). The evaporation mask defined a device area of 0.1 cm^2^.

Device performance was evaluated under a Class A solar simulator (ABET Technologies, Sun2000, Milford, CT, USA) at AM 1.5 G and 100 mW/cm^2^ connected with a sourcemeter (Keithley 2420); the irradiation level was verified at the same height and position where the solar cell is placed with a calibrated pyranometer (Skye SKS1110). The samples were covered during the measurement with a shadow mask with an opening area of 0.1 cm^2^ to illuminate only one cell at a time. A customized tool comprising a sourcemeter (Keithley 2612) and a monochromator (Newport 74000, Irvine, CA, USA) was used to measure the incident-photon-to-current conversion efficiency (IPCE) values of the device.

## 3. Results and Discussion

Aiming at choosing the best grid geometry for the realization of the semitransparent MLG/grid electrode, we tested several grid square dimensions evaluating the transmittance and sheet resistance. The results are reported in Table S1. Higher optical transmittance can be obtained obviously when the grid-spacing is increased, while low sheet resistance is obtained when the grid-spacing is decreased. To further investigate the trade-off between electrical and optical properties, we considered the ratio between the optical conductivity (δop) and the direct current conductivity (δdc) using the Tinkham’s Formula [41]:

(1) $$T(\lambda )={\left(1+\frac{Zo}{2Rs\;}\;\frac{\delta \mathrm{op}\;\left(\lambda \right)}{\delta \mathrm{dc}}\right)}^{-2}$$

where T(λ) is the transmittance, *Zo* is the free space impedance (377 Ω). Higher values of δdc/δop correspond to enhanced electro-optical performance.

The best balance between transmittance and sheet resistance was found for a 75 µm × 75 µm square dimension showing the 79.8% of transmittance and 165 Ω/□ of sheet resistance and for 150 µm × 150 µm square dimension with 5 µm of width, showing the 86% of transmittance and 355 Ω/□ of sheet resistance.

**Figure 4:**
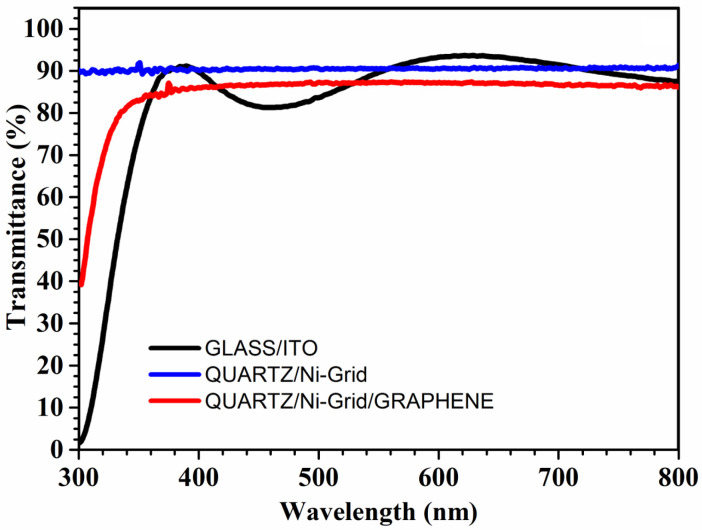
Optical transmission spectra, in the visible range, of quartz/Ni-grid (**blue line**) and quartz/Ni-grid/graphene (**red line**). For comparison the spectrum of ITO on glass is also included.

The optimum grid was then used for the transferring of the graphene layer. Figure 4 shows a comparison of the optical transmittance spectra of glass/ITO, quartz/Ni-grid, and quartz/Ni-grid/graphene films. As known, ITO exhibits a transmittance of 85.4% at 550 nm and a transparency modulation over a wide range of wavelengths, which is a drawback for ITO in optoelectronics due to non-uniform light absorption [42]. On the other hand, graphene on Ni-grid films shows a transparency of 83.2% at 550 nm, which is practically constant between 350 nm to 800 nm. The bare Ni-grid film shows a transparency of 86.2% at 550 nm, which is obviously slightly higher than the stack graphene/Ni-grid. The constant transparency of the graphene films on Ni-grid is an advantage for their applications in optoelectronics, allowing the uniform transmission of light in the whole wavelength range of interest.

**Figure 5:**
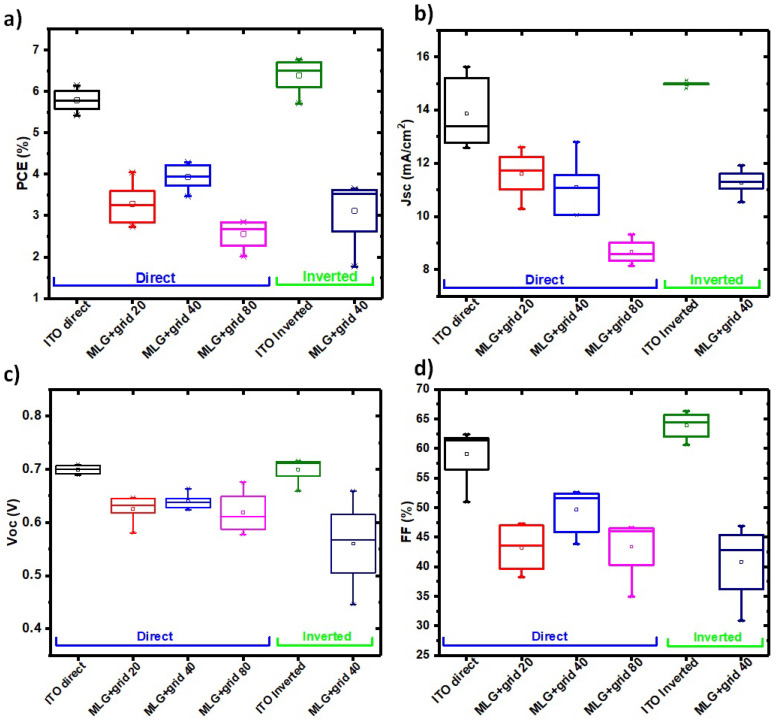
Tatistics of the J-V characteristics of the BHJ-SCs realized with direct and inverted architecture: (**a**) efficiency, (**b**) short circuit current, (**c**) open circuit voltage, (**d**) fill factor. Two different anodes have been tested: ITO/glass, and Graphene/Ni-grid on quartz. For the direct architecture different grid thicknesses have been tested (20 nm, 40 nm, 80 nm), for the inverted architecture only optimized thickness of 40 nm has been used.

The optimized graphene/Ni-grid geometry was then used as an electrode in OSCs: three different thicknesses of the grids, 20 nm, 40 nm and 80 nm were tested for the direct architecture, while the 40 nm thick grid, which was giving the best performances on the direct architecture, was chosen for the inverted one. The produced devices were compared with identical ones fabricated on glass/ITO, on bare Ni grids on quartz and monolayer graphene (MLG) on glass for both architectures. The measures on the MLG/Ni-grids have been performed on eight samples of each type, the resulting statistics are reported in Figure 5 for both architectures.

The best results for the direct architecture as function of the different anodes and thicknesses of the grid are reported in Table 1.

As expected, the performance of the devices realized on Ni-grid on quartz-based devices (Jsc = 2.2 mA/cm^2^, Voc = 633 mV, FF = 40.2% and PCE = 0.5%) and only graphene (Jsc = 4.7 mA/cm^2^, Voc = 580 mV, FF = 26% and PCE = 0.7%) were lower than the most efficient Ni-grid/MLG based device (Jsc = 12.7 mA/cm^2^, Voc = 638 mV, FF = 51.9% and PCE = 4.2%). The primary differences were in short circuit current (Jsc) and fill factor (FF); the decrease in Jsc reflected the lower collection charge of the Ni-grid due to the absence of a continuous anode, while, in the case of the MLG graphene, it could be ascribed to the defects in the graphene layer induced by the transferring method [33]. For the MLG, the high sheet resistance in the range of 900 Ω/□ strongly affected the FF which, reached only 26.0% (see Table 2). Comparing the different Ni-Grid/MLG-based solar cells, it emerges that the sheet resistance was not the only parameter that affected the final performances of the devices. In fact, the 80 nm thick grid performed worse than the 40 nm grid despite the better sheet resistance. This is related to the height of the grid that, being comparable with the thickness of the deposited active layer, induced a worse deposition of the different layers of the solar cells, influencing the overall PCE. In case of 40 nm thick layers instead, the deposition of the different layers was quite uniform, as can be seen in the image reported in the supporting info Figure S1.

**Figure 6:**
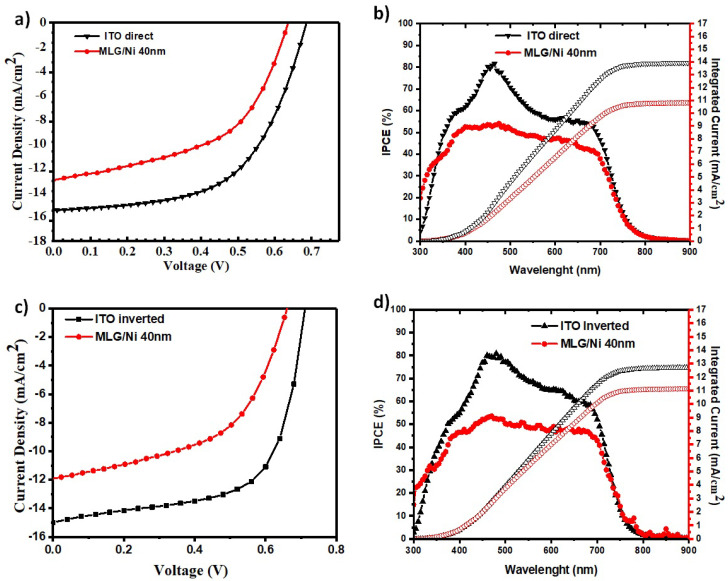
J-V characteristics of the bulk heterojunction solar cells on two different anodes: ITO/glass, and Graphene/Ni-grid 40 nm/quartz for direct (**a**) and inverted (**c**) architecture. IPCE spectrum for direct (**b**) and inverted (**d**) OSCs. The right-hand axis indicates the integrated photocurrent that is expected to be generated under AM 1.5G irradiation on two different anodes: ITO/glass and Graphene/Ni-grid/quartz.

On the substrate with the optimized 40 nm thick Ni-Grid, we realized also organic solar cells with the inverted architecture. The electrical characterization of the best OSC realized with both configurations were compared with the one obtained on glass-ITO substrates and reported in Figure 6a, together with the measured IPCE (Figure 6b), while the comparison of the best solar cells obtained with the direct architecture varying the grid thickness are reported in the Figure S2.

In the case of direct architecture, the best performance of the solar cells obtained for MLG/Ni-grid (40 nm)-based devices (Jsc = 12.7 mA/cm^2^, Voc = 638 mV, FF = 51.9% and PCE = 4.20%), showed a reduction in efficiency of about 30% when compared with the ITO-based devices (Jsc = 15.6 mA/cm^2^, Voc = 689 mV, FF = 57.2% and PCE = 6.1%). Figure 6b shows the IPCE spectrum of the two OSCs. Generation of photocurrent in this case started at 700 nm, in agreement with the band-gap of the PTB7:PCBM active layer, and showed an excellent photocurrent response until 400 nm. The integration of the IPCE spectrum with the AM 1.5G solar photon flux, yielded to a current density of 13.9 mA/cm^2^ and 10.8 mA/cm^2^, respectively, on ITO/glass and MLG/Ni-grid/quartz anodes.

Comparing the performance of these two types of solar cells, it is evident that the sheet resistance was the bottle neck for the MLG/Ni-grid based devices; this is reflected by the decreased FF due to higher series resistance. This is evidenced also by the J-V current measures done in dark and reported in Figure S3, where it is possible to notice that both devices had a rectifying behavior in the forward part of the characteristic. Comparing the two curves, the ITO-based device shows for voltages above 0.7 V a steeper slope than the MLG/Ni grids-based devices that can be ascribed to a lower series resistance that can be ascribed to the lowest sheet resistance of the ITO contact. The MLG/Ni grids-based devices showed also a higher leakage current compared to the ITO based devices that reflected lower shunt resistance. This could be ascribed to the interfacial defects present in between the different layers of the solar cells or on the inhomogeneity of the different deposited layer [43].

In addition to that, we can observe a slight difference in the Jsc due to the lower transmittance spectrum of the cells with graphene/Ni-grid anode, as reported in Figure 4.

Similar results were obtained from inverted devices whose best performances are reported in Figure 6c,d and summarized in Table 2.

**Figure 7:**
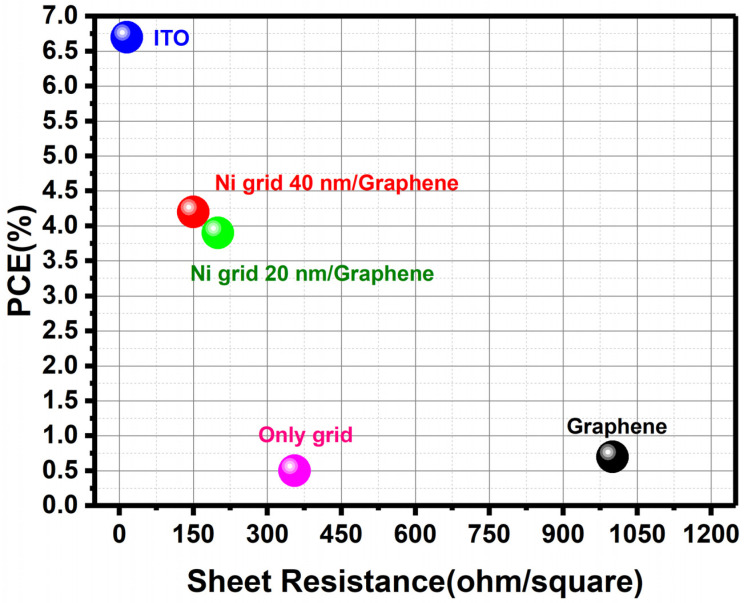
Summary of the best performance obtained with the organic solar cells realized with the different type of transparent conductive electrodes reported in this work.

In this case, we observed not only a decrease of FF for the MLG/Ni- Grid devices, but also a reduction in the Voc. This could be due to the non-uniform deposition of the PEIE layer. This material, which normally has a thickness in the range of few nm, was deposited via spin coating on a substrate, where the grid introduced a periodical repetition of highs and valleys that resulted in a roughness that could have affected the overall deposition. The repercussions are visible in Voc and at the end, on the final efficiency, which, in this case, is 45% lower than the reference. A summary of the best efficiencies achieved with the different type of transparent conductive electrodes considered in this work is reported in Figure 7.

## 4. Conclusions

In conclusion we have fabricated conductive and transparent films using commercially available monolayer graphene synthesized by thermal CVD and transferred on Ni-grids. The technological steps to realize a Ni-Grid with a specific geometric profile able to reduce the cracks in the graphene film have been reported and the combination of the grid with the transferred graphene layers allowed to obtain semitransparent electrodes with a maximum transmittance of 90% (calculated in 300–800 nm range) and a sheet resistance down to 35 Ω/□. The hybrid transparent Ni-grid/MLG films were then employed as semitransparent devices for the realization of PTB7:PCBM organic solar cells, yielding a maximum PCE of 4.2% in direct architecture. The OSCs with the hybrid anodes exhibited significantly better performance in comparison with the ones realized with only MLG and raw Ni-grid. However, when compared with the reference cells based on ITO they demonstrated a 30% and 46% reduction in efficiency with a direct and inverted architecture respectively. These results can be ascribed mainly to the higher sheet resistance of the Ni-grid/MLG electrodes that cause a decrease in the FF of the solar cells and also to the defects at the interfaces between the semitransparent electrodes and the solar cells layers. Further improvements in terms of efficiency of the devices could be achieved reducing the contamination of the graphene layer during the transfer on the grids reducing the effects of the parasitic resistances of the devices, in addition to the introduction of proper doping of the graphene layer. Considering the realization of organic solar modules, the introduction of the grids would be of paramount importance when aiming to scale up to large area devices. Their geometry will be of fundamental importance to guarantee uniform deposition of the different layers, allowing one to obtain working devices and representing a possible route towards the realization of a more sustainable, ITO-free organic solar cells and modules.

## Figures and Tables

**Table 1:** Photovoltaic parameters of BHJ solar cells in direct configuration on glass-ITO (reference), MLG/Ni-grid with different thicknesses, pristine Ni-grid and MLG only as anodes, fabricated in the same batch with 0.1 cm^2^ of active area.

Anodes	Sheet Resistance (Ω/□)	Voc (mV)	Jsc (mA/cm^2^)	FF (%)	PCE (%)
ITO	~15	689	15.6	57.2	6.1
Ni-grid (40 nm)	~355	633	2.2	40.2	0.5
MLG	~900	580	4.7	26.0	0.7
MLG/Ni-grid (20 nm)	~200	646	12.6	47.3	3.9
MLG/Ni-grid (40 nm)	~150	638	12.7	51.9	4.2
MLG/Ni-grid (80 nm)	~35	675	9.1	45.6	2.8

**Table 2:** Photovoltaic parameters of BHJ solar cells in inverted configuration on glass-ITO (reference), graphene/Ni-grid with different thicknesses as cathode, fabricated in the same batch with 0.1 cm^2^ of active area.

Anodes	Sheet Resistance (Ω/□)	Voc (mV)	Jsc (mA/cm^2^)	FF (%)	PCE (%)
ITO	~15	712	15,1	63.2	6.7
Ni-grid (40 nm)	~355	554	1.7	42.1	0.4
MLG	~900	405	3.5	35.2	0.5
MLG/Ni-grid 40 nm	~150	659	11.9	46.5	3.6

## Data Availability

The data presented in this study are available on request from the corresponding author.

## Supplementary Materials

The following supporting information can be downloaded at: https://www.mdpi.com/article/10.3390/polym14051046/s1, Figure S1: Deposition of MoO_3_/PTB7 on Glass/ITO (a), Quartz/MLG (b) and Quartz/Ni-Grid/MLG (c); Figure S2: Best performing solar cells realized with direct architecture varying the Ni grid thickness: (a) J-V characteristics (b) IPCE spectrum; Figure S3: Comparison of J-V characteristics measured in dark for the best solar cells realized on ITO and MLG/Ni Grid (40 nm) for direct architecture (a) and inverted architecture (b); Table S1: State of the art compared to the results reported in this paper.
