# Supplementary material for: Graphene with Ni-Grid as Semitransparent Electrode for Bulk Heterojunction Solar Cells (BHJ-SCs)
Source: Polymers (Basel). 2022 Mar 5;14(5):1046. doi: 10.3390/polym14051046 (PMC8915009; doi:10.3390/polym14051046)
Supplement: Supplementary file 1 [file polymers-14-01046-s001.zip › polymers-1557755-supplementary.pdf]

polymers

Supplementary

Graphene with Ni-grid as semitransparent electrode for bulk heterojunction solar cells (BHJ-SCs)

Martina Dianetti[1], Gianpaolo Susanna[1,2], Emanuele Calabrò [1], Giuseppina Polino[1], Martin Otto[3], Daniel Neumaier [3,4], Andrea Reale[1], Francesca Brunetti[1]*

1 Department of Electronic Engineering, University of Rome Tor Vergata—CHOSE, Via del Politecnico 1, 00133 Rome, Italy; martina.dianetti@uniroma2.it (M.D.); gianpaolo.susanna.ext@mise.gov.it (G.S.); emanuele.calabro@uniroma2.it (E.C.); giuseppina.polino@uniroma2.it (G.P.); reale@ing.uniroma2.it (A.R.)
2 ISCTI—Istituto Superiore delle Comunicazioni e delle Tecnologie dell'Informazione-Ministero dello Sviluppo Economico, Viale America 201, 00144 Rome, Italy
3 Gesellschaft für Angewandte Mikro-und Optoelektronic mBH—AMO GmbH, Otto-Blumenthal-Straße 25, 52074 Aachen, Germany; otto@amo.de (M.O.); neumaier@amo.de (D.N.)
4 Chair of Smart Sensor Systems, Bergische University of Wuppertal, 42119 Wuppertal, Germany
* Correspondence: francesca.brunetti@uniroma2.it; Tel.: +39-0672597366

1. Tables

Table S1. Values of sheet resistance and transmittance at 550 nm of Ni-grid on quartz with different dimensions and the ratio between direct current conductivity (δdc) and optical conductivity (δop). Mean and maximum value measured in the range between 300 nm and 800 nm.

Ni-Grid Square (µm)	T (%) @550 nm	T (%) Mean btw 300–800 nm	Sheet Resistance (Ω/□)	δ_{dc}/δ_{op}
75	79.8	79.6	~165	9.5
150	86.2	86.3	~355	6.8
200	88.1	88.2	~465	6.2
300	90.0	90.1	~780	4.4

Table S2. State of the art compared to the results reported in this paper.

Type of Graphene Electrode	Solar Cell Layers	Sheet Resistance (Ω/□)	Voc (mV)	Jsc (mA/cm²)	FF (%)	PCE (%)	Ref.
Functionalized solution processed graphene	CuPc/C60/BCP/ Ag	500K	0.5	2.1	34	0.4	[1]
MLG	PEDOT:PSS/P3HT:PCBM/Ca/Al	~370	0.5	6.9	32	1.2	[2]
P-Doped MLG	MoO3/PEDOT:PSS/P3HT:PCBM/LiF/Al	~100	0.6	7.8	51	2.5	[3]
P-Doped MLG	ZnO/PEIE/ PffBT4T-2OD:PCBM/ V_2O_5/Ag	30	0.7	10.5	46	2.8	[4]
P-Doped MLG	PEDOT:PSS/PTB7:PCBM/ MoO_3/Ag	25	0.7	13.8	41	4.1	[5]
Flexible P-Doped MLG	PEDOT:PSS/P3HT:PCBM/ZnO/Doped MLG	~200	0.6	9.8	54	3.3	[6]
Flexible P-Doped MLG	PEDOT:PSS/PM6:Y6/PDINO/Al	83	0.8	25.8	70	15.2	[7]
MLG/Ni-grid	MoO_3/PTB7:PCBM/Ca/Al	~150					This Work
MLG/Ni-grid	PEIE/PTB7:PCBM/ MoO_3/Ag	~150					This Work

2. Figures

a) b) c)

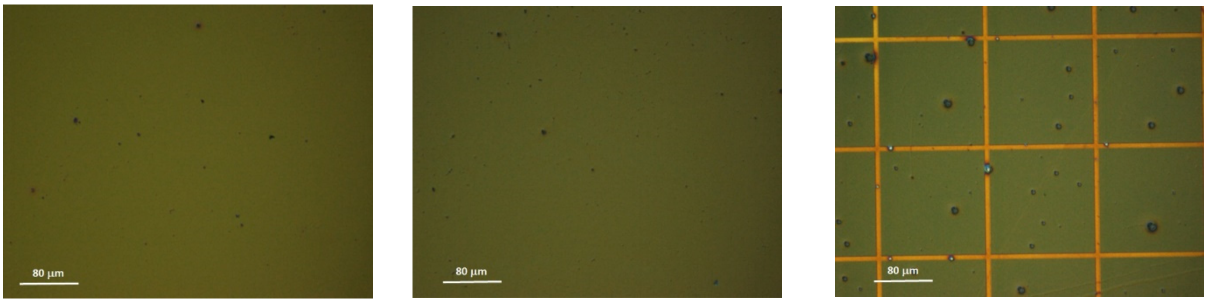

Figure S1. Deposition of MoO3/PTB7 on Glass/ITO (a), Quartz/MLG (b) and Quartz/Ni-Grid/MLG (c).

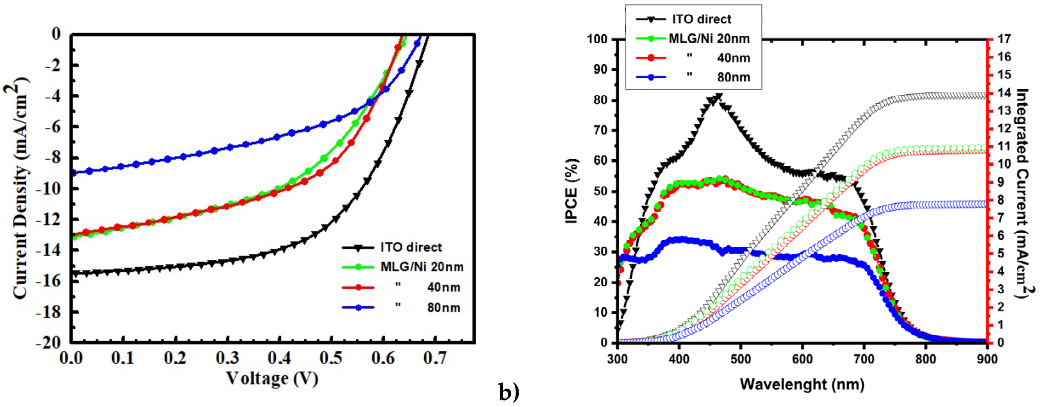

a) b)

Figure S2. Best performing solar cells realized with direct architecture varying the Ni grid thickness: (a) J-V characteristics (b) IPCE spectrum.

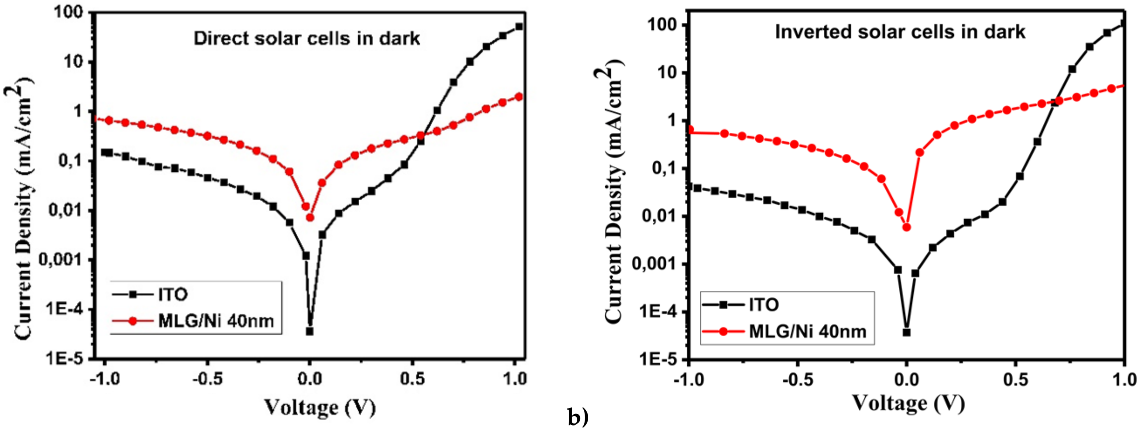

a) b)

Figure S3. Comparison of J-V characteristics measured in dark for the best solar cells realized on ITO and MLG/Ni Grid (40 nm) for direct architecture (a) and inverted architecture (b).

References

1. Wu J, Becerril HA, Bao Z, Liu Z, Chen Y, Peumans P. Organic solar cells with solution-processed graphene transparent electrodes. Applied Physics Letters. 2008;92:263302.
2. Choi Y-Y, Kang SJ, Kim H-K, Choi WM, Na S-I. Multilayer graphene films as transparent electrodes for organic photovoltaic devices. Solar Energy Materials and Solar Cells. 2012;96:281-5.
3. Wang Y, Tong SW, Xu XF, Özyilmaz B, Loh KP. Interface Engineering of Layer-by-Layer Stacked Graphene Anodes for High-Performance Organic Solar Cells. Advanced Materials. 2011;23:1514-8.

4. La Notte L, Villari E, Palma AL, Sacchetti A, Michela Giangregorio M, Bruno G, et al. Laser-patterned functionalized CVD-graphene as highly transparent conductive electrodes for polymer solar cells. Nanoscale. 2017;9:62-9.
5. La Notte L, Bianco GV, Palma AL, Di Carlo A, Bruno G, Reale A. Sprayed organic photovoltaic cells and mini-modules based on chemical vapor deposited graphene as transparent conductive electrode. Carbon. 2018;129:878-83.
6. Shin DH, Jang CW, Lee HS, Seo SW, Choi S-H. Semitransparent Flexible Organic Solar Cells Employing Doped-Graphene Layers as Anode and Cathode Electrodes. ACS Applied Materials & Interfaces. 2018;10:3596-601.
7. Koo D, Jung S, Seo J, Jeong G, Choi Y, Lee J, et al. Flexible Organic Solar Cells Over 15% Efficiency with Polyimide-Integrated Graphene Electrodes. Joule. 2020;4:1021-34.
